# Hydrological Modeling of the Jiaoyi Watershed (China) Using HSPF Model

**DOI:** 10.1155/2014/672360

**Published:** 2014-06-09

**Authors:** Chang-An Yan, Wanchang Zhang, Zhijie Zhang

**Affiliations:** ^1^State Key Laboratory of Pollution Control & Resource Reuse, School of the Environment, Nanjing University, Nanjing 210093, China; ^2^Key Laboratory of Digital Earth Science, Institute of Remote Sensing and Digital Earth (RADI), Chinese Academy of Sciences (CAS), Beijing 100094, China; ^3^College of Automation, Nanjing University of Posts and Telecommunications, Nanjing 210046, China

## Abstract

A watershed hydrological model, hydrological simulation program-Fortran (HSPF), was applied to simulate the spatial and temporal variation of hydrological processes in the Jiaoyi watershed of Huaihe River Basin, the heaviest shortage of water resources and polluted area in China. The model was calibrated using the years 2001–2004 and validated with data from 2005 to 2006. Calibration and validation results showed that the model generally simulated mean monthly and daily runoff precisely due to the close matching hydrographs between simulated and observed runoff, as well as the excellent evaluation indicators such as Nash-Sutcliffe efficiency (NSE), coefficient of correlation (*R* ^2^), and the relative error (RE). The similar simulation results between calibration and validation period showed that all the calibrated parameters had a certain representation in Jiaoyi watershed. Additionally, the simulation in rainy months was more accurate than the drought months. Another result in this paper was that HSPF was also capable of estimating the water balance components reasonably and realistically in space through the whole watershed. The calibrated model can be used to explore the effects of climate change scenarios and various watershed management practices on the water resources and water environment in the basin.

## 1. Introduction

Since the 20th century, the pattern of earth's water cycle has changed significantly due to the increasing enhancement of global climate change and human activities. Water resources and water environment problems of watershed, such as the intensification of spatial-temporal change of water resources [1, 2], the frequent occurring of extreme droughts and floods weather [3], and the deterioration of water quality [4, 5], directly threaten human health and affect sustainable development of the economy and society [6, 7]. Watershed model, widely used to simulate watershed-scale water cycle processes and evaluate the hydrological response to various management strategies, has become one of the most powerful tools for watershed management in the last two decades [8]. It is an effective tool for assessing the effect of climate and land use change and water resources management, as well as disaster prevention and mitigation in a watershed.

The flood disaster occurs frequently and water shortage in China has become a big issue, not only because of the inaccuracy for runoff prediction, but also because of the imbalance between available scant water resources and population. As an important tributary on the upper reaches of the Huaihe River, the biggest river in the eastern China, the water resources and water environment of the Benghe River will definitely affect the South-North Water Diversion Project. It is, therefore, essential to introduce watershed hydrological model for Jiaoyi watershed so as to predict runoff accurately and clarify the spatial and temporal variation of water resources in Jiaoyi watershed.

There are numerous distributed watershed hydrological models that can continuously simulate runoff from watershed; some of the most important and widely used ones are TOPMODEL (topography based hydrological model) [9], SWAT (soil and water assessment tool) [10], SHE (système hydrologique Européen) [11], and HSPF (hydrologic simulation program-Fortran) [12]; all models have different applicability and limitations for watershed. In China, SWAT is the most popular watershed hydrological model and applications of SWAT have expanded all over the country over the past decade. HSPF model, which uses an infiltration-excess mechanism to simulate streamflow [13], has been successfully applied to a number of watersheds in United States and European Union. However, there has been little research on simulating runoff using HSPF in the semidry and semihumid climatic conditions of China. Therefore, the aim of this paper is to simulate the spatial and temporal variation of hydrological processes and evaluate the performance of the HSPF model in the Jiaoyi watershed of Huaihe River Basin.

## 2. Materials and Methods

### 2.1. Study Area

**Figure 1:**
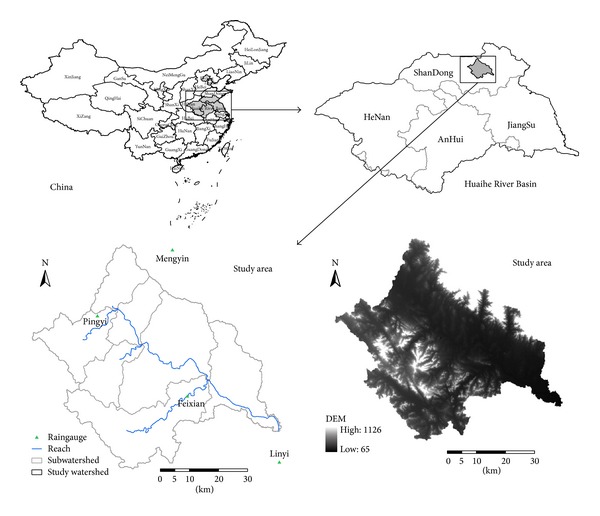
Geographical location of study area within Huaihe River Basin, China.

Benghe River, the main tributary in Yihe River systems of the Huaihe River Basin, originates from the Taihang Mountain in Pingyi, Shandong province, drains an extensive area with river channel about 158 km in length and flows across Feixian, Linyi counties, and at last discharges into the Yihe in the north of Linyi city. The selected watershed in this study is located in the upstream section of the Benghe, controlled by the Jiaoyi Hydrometric Station along the Benghe River, and it is referred to as the Jiaoyi watershed. The total drainage of this watershed is about 3366 km^2^. The elevation varies between 75.3 and 1026 m. The average stream gradient is about 0.73‰, and the low mountainous area and hill area cover about 41% and 59% of the study watershed, respectively. It has typical temperate continental monsoon climate with an annual average temperature of 13.9°C and an annual average precipitation of 819.3 mm. Rainfall shows high seasonal variability, with about 75% of annual precipitation falling from July to September. It is also a typical semimoisture semiaridity region; most of the watershed area is covered by forest land, and the other land use types are agricultural land, urban or built-up land, and wetlands/water. The dominant soils of the watershed are cinnamon soil, brown soil, moisture soil, and Shajiang black soil. See Figure 1 for a map of the study area.

### 2.2. Description of HSPF Models

HSPF model, which has been developed to simulate water quantity and quality at any point in the watershed by U.S. Environmental Protection Agency (USEPA), is a distributed, continuous time watershed scale model evolved from the Stanford watershed model (SWM) which is the watershed hydrological model in which the mathematics methodology is applied to hydrological calculation and forecasting. HSPF model is composed of three application modules: PERLND, IMPLND, and RCHRES [14]. PERLND and IMPLND modules simulate the hydrologic and water quality processes over pervious land surfaces and impervious land surfaces, respectively, and the RCHRES module is utilized to represent hydraulic and water quality processes for streams and well-mixed lakes and reservoirs [15]. Water quality constituents simulated by HSPF include temperature, dissolved oxygen, biochemical oxygen (BOD), sediment detachment and transport, sediment routing, nitrate, organic nitrogen, organic phosphorus, orthophosphate, ammonia nitrogen, pesticides, conservatives, phytoplankton, and zooplankton [16], and the hydrologic components of HSPF are simulated by five storage classes (interception, upper zone, lower zone, base flow, and deep percolation) in which each allows different types of inflow and outflow; all inflows and outflows are based on the principle of water balance; the following processes such as interception, evapotranspiration, surface detention, surface runoff, infiltration, shallow subsurface flow (interflow), base flow, and deep percolation [17] occur in each pervious land segment (hydrologic response unit). HSPF applies Manning's equation for routing overland flow and kinematic wave method for channel routing [18]. Detailed information about the structure and theories of HSPF can be found in the HSPF version 12 user's manual [14]. HSPF requires extensive data input and complex procedure in initial stage; for user's convenience, the USEPA has developed the better assessment science integrating point and nonpoint sources (BSINS) [19], which is watershed management system based on GIS, integrating these hydrological models such as HSPF, SWAT, PLOAD, and AGWA, as well as auxiliary means WDMUtil and GenScn. The major discrepancies approaches adopted for representing different hydrological processes between SWAT and HSPF are summarized in Table 1.

### 2.3. Model Input Data Descriptions

HSPF model requires the input data including digital elevation model (DEM), land use, soil data, and meteorological data. The DEM for the study area, which has a 30 × 30 m horizontal resolution, was downloaded from the International Scientific Data Service Platform, Chinese Academy of Science. Referring to the land use data with 1 km resolution in China from Institute of Geographical Sciences and Natural Resource Research, Chinese Academy of Sciences, the land use data in this paper were acquired by the method of supervised classification based on remote sensing Landsat ETM images. The soil data were downloaded from the Data Center for Resources and Environmental Sciences, Chinese Academy of Sciences. The meteorological data for simulation (2000–2007) were collected from six weather stations (Figure 1) nearest to the study watershed. In HSPF, the required climate data such as precipitation, evapotranspiration, air temperature, wind speed, solar radiation, dew-point temperature, and cloud cover are inputted by hourly time step, and these climate data were stored in the watershed data management (WDM) program from BASINS.

Besides those data mentioned above, SWAT and HSPF also need hydrological time series of flow for model calibration and validation. The daily observed flow data for the period from 1/1/2001 to 31/12/2006 were obtained from Jiaoyi Hydrometric Stations.

### 2.4. Model Calibration and Validation

Model calibration is a process of adjusting model parameters within a suitable range to achieve agreement between observed and simulated flows, while model validation is a process of evaluating the calibrated model parameters to determine the most matching model parameters. HSPF were calibrated for the period from 1/1/2001 to 31/12/2004 and then validated from 1/1/2005 to 31/12/2006.

In HSPF, automatic Parameter Estimation software (PEST) [20] aided by HSPEXP is applied to calibrate HSPF model, and 9 HSPF parameters, the most sensitive to the simulation of hydrologic process in HSPF [21], were adjusted to acquire a match between simulated and observed flows during the period of calibration and validation.  Table 2 lists the description of these parameters together with their values calibrated in HSPF.

### 2.5. Evaluation Criteria

The monthly and daily runoff simulated by SWAT and HSPF model were quantitatively evaluated by coefficient of determination (*R* ^2^), Nash-Sutcliffe coefficient (NSE), and relative error (RE), which are defined as follows:

(1) $$\begin{array}{c} R^{2}=\frac{{\left[\sum_{i=1}^{n}\left(y_{\text{sim}}^{i}-\bar{y_{\text{sim}}}\right).\left(y_{\text{obs}}^{i}-\bar{y_{\text{obs}}}\right)\right]}^{2}}{\sum_{i=1}^{n}{\left(y_{\text{sim}}^{i}-\bar{y_{\text{sim}}}\right)}^{2}.\sum_{i=1}^{n}{\left(y_{\text{obs}}^{i}-\bar{y_{\text{obs}}}\right)}^{2}},\\ \text{NSE}=1-\frac{\sum_{i=1}^{n}{\left(y_{\text{obs}}^{i}-y_{\text{sim}}^{i}\right)}^{2}}{\sum_{i=1}^{n}{\left(y_{\text{obs}}^{i}-\bar{y_{\text{obs}}}\right)}^{2}},\\ \text{RE}=\frac{\sum_{i=1}^{n}\left(y_{\text{sim}}^{i}-y_{\text{obs}}^{i}\right)}{\sum_{i=1}^{n}y_{\text{obs}}^{i}}\times 100\%, \end{array}$$

where *y*_sim_^*i*^ is daily simulated flow in *i* day, *y*_obs_^*i*^ is daily observed flow in *i* day, $\bar{y_{\text{obs}}}$ is average observed flow of simulated period, $\bar{y_{\text{sim}}}$ is average simulated flow of simulated period, and *i* is day.

Both NSE and *R* ^2^ indicate the consistency with which simulated values versus observed values follow a best fit line; their values close to 1 demonstrate perfect fit, while RE, whose value close to 0 shows perfect fit, describes the difference between model simulations and observations in the units of the variable. In this study, the general performance ratings, as modified by Moriasi et al. [22] and Parajuli et al. [23], were used to judge the model performance, as Table 3 shows.

## 3. Results and Discussion

**Figure 2:**
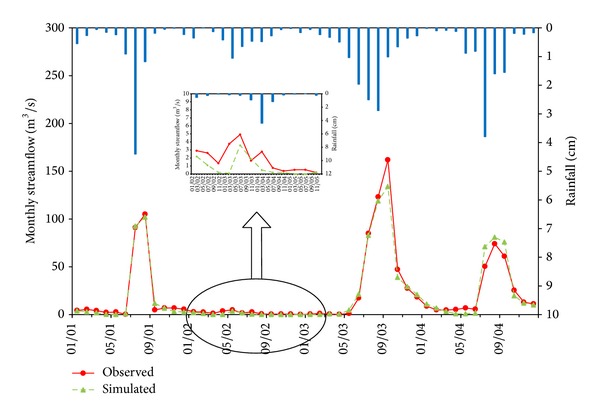
Comparison of observed and simulated results for mean monthly rainfall-runoff simulation in Jiaoyi watershed during calibration period (2001~2004).

**Figure 3:**
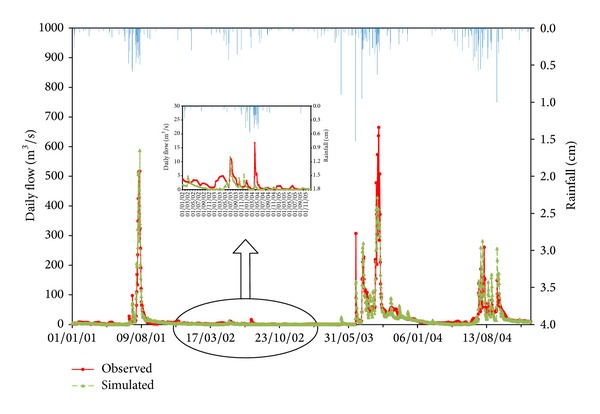
Comparison of observed and simulated results for daily rainfall-runoff simulation in Jiaoyi watershed during calibration period (2001~2004).

**Figure 4:**
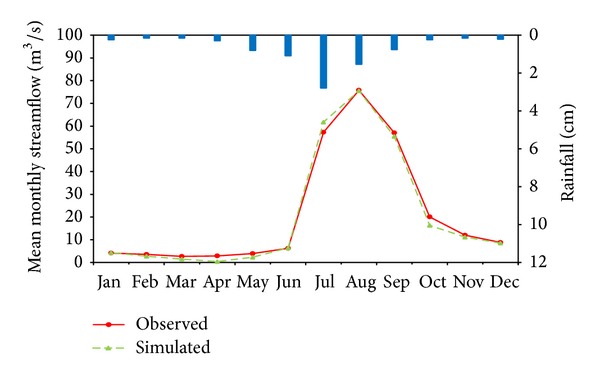
Comparison of observed and simulated results for monthly mean rainfall-runoff simulation in Jiaoyi watershed during calibration period (2001~2004).

**Figure 5:**
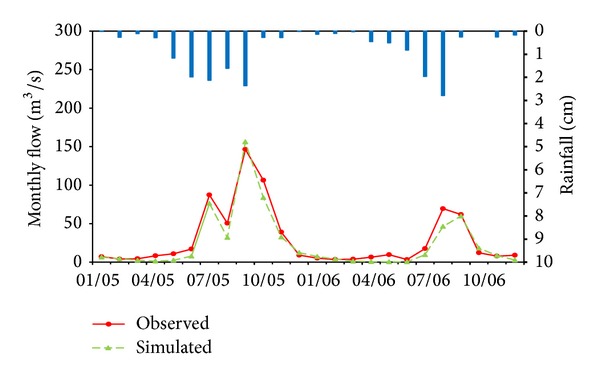
Comparison of observed and simulated results for mean monthly rainfall-runoff simulation in Jiaoyi watershed during validation period (2005~2006).

**Figure 6:**
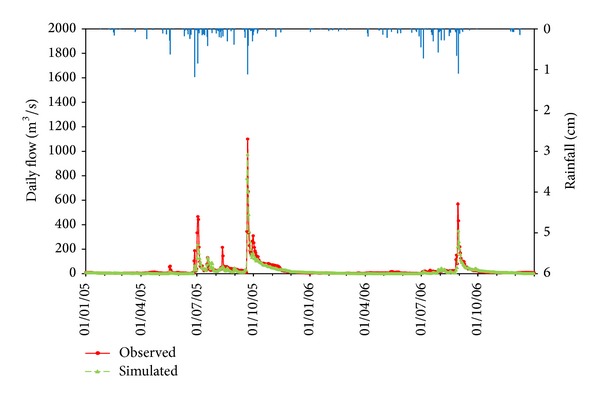
Comparison of observed and simulated results for daily rainfall-runoff simulation in Jiaoyi watershed during validation period (2005~2006).

The simulated monthly mean and daily runoff were plotted with the observed values during the calibration and validation period (Figures 2, 3, 4, 5, and 6). Statistics resulting from monthly mean and daily runoff simulation for model calibration and validation were shown in Table 4, while Table 2 presented the calibrated values of model parameters.

### 3.1. Model Calibration Results

During the calibration period, the mean monthly and daily runoff hydrographs for the 4-year simulation period display the acceptable agreement and tendency. The peak flows were shown in the same time between simulated and observed flow hydrograph, but the simulated peak flows were slightly higher than observed peaks during the flood season of 2001 and 2004 and lower in 2002 and 2003. In general, whether from the time to peaks or from the peak values, the simulation results in drought years (2002) were much worse than those in wet years, and the simulation results with single peaks (2001) performed better than multipeaks (2003 and 2004) through the whole wet years. It may imply that HSPF is sensitive to the amount of rainfall.

Statistical indicators from Table 4 also quantitatively confirm the above analyses. The NSE and *R* ^2^ for mean monthly runoff during the calibration period (2001–2004) both were 0.97 and for the daily runoff they both were 0.86. Meanwhile, compared with observed mean monthly and daily runoff volumes, HSPF underestimated mean monthly and daily runoff volumes with 3.0% and 2.1% relative error, respectively. Therefore, it was concluded that HSPF simulated both mean monthly and daily runoff excellently on the basis of Table 3. Additionally, the average relative error analysis for monthly mean runoff in calibration period (2001–2004) was used to present seasonal variation of runoff simulation, as displayed in Table 5. From Table 5 and Figure 4 it can obviously be seen that the simulated mean monthly runoff has the smallest average relative error during the rainy season (6–9), while the largest average relative error with underestimated mean monthly runoff occurs in the spring months (2–5). This phenomenon also verifies that the runoff volume simulated by HSPF has a good response to rainfall. In other words, the simulated runoff volume closely matched observed values in the rainy season. The possible cause of this phenomenon is that the rainfall did not immediately generate runoff but primarily replenished soil moisture due to the lower soil moisture content after the long drought. The model parameters may result in the lower simulation performance in the drought season. In HSPF, parameters LZSN, INFILT, AGWRC, INTFW, and IRC, which mainly control the water balance all over the watershed, were fixed throughout the calibration period in both drought and rainy seasons. This can cause that runoff generation may have formed too fast in rainy season, however the runoff generation may have not occurred quickly enough in drought season. Therefore, this paper suggested that different model parameters for drought and rainy seasons may improve model simulation efficiency in drought.

### 3.2. Model Validation Results

During the validation period, the hydrographs (Figures 5 and 6) of simulation and observed runoff show that model in general is able to reproduce the entire shape of hydrographs perfectly. Although the simulated peak runoff and the time to peak matched well with the observed runoff, all the peaks in daily runoff simulation were underestimated. Moreover, the daily runoff simulation with single peaks (2006) performed better than multipeaks (2005) through the whole wet years. These descriptions were confirmed by the evaluation criteria in Table 4, which indicated that model was excellent to simulate the mean monthly runoff due to excellent agreement and correlation (NSE = 0.94 and *R* ^2^ = 0.96). Furthermore, the daily NSE = 0.80 and *R* ^2^ = 0.81 values obtained indicated that model performance was “very good” in simulating daily runoff for this validation period. However, the relative errors of mean monthly and daily runoff estimated by HSPF were both considered “good” with −17.3% RE and −17.5% RE, respectively. Table 4 also revealed that the simulation accuracy was very similar in the calibration and validation periods. It should be concluded that all the calibrated parameters (Table 2) have a certain representation in Jiaoyi watershed. Meanwhile, HSPF could simulate the hydrological process excellently and stably.

### 3.3. Spatial Analysis of Hydrological Process in Jiaoyi Watershed

As an excellent distributed watershed hydrological model, HSPF can output the overall water balance components including rainfall, runoff (surface runoff, interflow, and base flow), evaporation losses (potential, interception, upper zone, lower zone, base flow, and active groundwater), and deep groundwater recharge/losses for individual land uses. It is very important to analyze the temporal and spatial variation of overall water balance components for comprehending the hydrologic processes in watershed. Besides, these water balance components will also help us insure the rationality of calibrated model parameters and make sure that they are logical and realistic.  Table 6 shows annual water balance components for agricultural land as an example. In Table 6 it is seen that annual interflow is the highest contribution of annual runoff for agricultural land and annual surface runoff is the lowest contribution of annual runoff in rainy year. While in drought year (2002) annual base flow contributes the most to annual runoff. The evaporation of upper zone accounts for the largest in actual evaporation in agriculture land, while the evaporation of ground water and base flow are the minimum. Potential evaporation in drought year is greater than rainy year because the great air humidity can reduce the evaporation of soil and vegetation in year of abundant rainfall.

**Figure 7:**
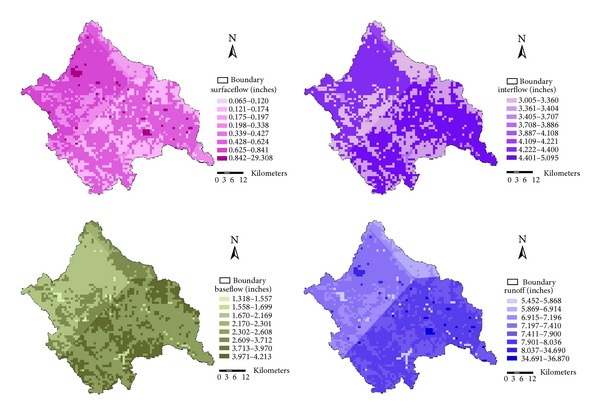
Spatial distribution of different hydrological simulation results for annual average between 2001 and 2007.

For better understanding the spatial distribution characteristics of water balance components in the whole Jiaoyi watershed, annual average surface flow, interflow, base flow, and runoff were displayed in Figure 7. It shows spatial negative correlation between base flow and interflow. The distribution pattern of base flow is relatively higher in hilly areas, where the interflow is lower. However, runoffs have high spatial correlation with interflow. This demonstrates that base flow is the major component of runoff. From Figure 7 we can see that runoff near the outlet is relatively higher with common sense. These preliminary conclusions show that HSPF has high accuracy and reliability of simulating the spatial distribution of water balance components.

## 4. Summary and Conclusions

In this study, watershed scale hydrological simulation model HSPF was employed to simulate hydrological behavior and spatial distribution of water balance components in Jiaoyi watershed located in eastern China. Results showed that HSPF model was excellent to simulate mean monthly and daily runoff adequately due to excellent agreement and correlation (*R* ^2^ = 0.97 and NSE = 0.97 for mean monthly simulation and *R* ^2^ = 0.86 and NSE = 0.86 for daily simulation) during the calibration period (2001–2004). With the same model parameters, it simulated mean monthly and daily runoff perfectly although the evaluation indicators drooped slightly in the validation period (2005-2006). It should be concluded that all the calibrated parameters have a certain representation in Jiaoyi watershed. Meanwhile, HSPF could simulate the hydrological process excellently and stably. Moreover, the discrepancies were found in runoff simulation for drought and rainy months. It showed the relative error of mean monthly runoff simulation in rainy months was much less than that in drought months. Therefore, this paper suggested that different model parameters for drought and rainy season may improve model simulation efficiency in drought. Finally, HSPF displayed the spatial distribution of annual average surface flow, interflow, base flow, and runoff in Jiaoyi watershed. Based on reasonable and logical spatial distribution characteristics, it is revealed that HSPF not only can simulate runoff precisely in time, but can also estimate the water balance components realistically in space. Thus, HSPF model capable of helping understand watershed hydrological cycle processes accurately can be utilized by watershed manages. The further research will use the HSPF model with calibrated parameters to investigate the effects of different watershed management and climatic scenarios on water resource and water environment at the watershed scale.

## Figures and Tables

**Table 1:** Summary of the approaches adopted in SWAT and HSPF for hydrological process simulations.

Model	Surface runoff	Infiltration	Channel routing	Snowmelt runoff	Compute unit
SWAT	Modified SCS curve	Green-Ampt	Muskingum	Degree-day method	HRU
HSPF	Chezy-Manning	Philip equation	Kinematic wave	Energy balance approach	Segment

**Table 2:** List of adjusted parameters for calibration of HSPF model.

Parameter	Definition	Unite	Calibrated value
LZSN^1^	Lower zone nominal soil storage	inches	2.000
LZSN^2^			4.819
LZSN^3^			2.000
INFILT^1^	Index to infiltration capacity	in/hr	0.315
INFILT^2^			4.835
INFILT^3^			0.140
AGWRC^1^	Groundwater recession rate	1/d	0.998
AGWRC^2^			0.973
AGWRC^3^			0.999
DEEPFR	Fraction of GW inflow to deep recharge	none	8.69*E* − 02
BASETP	Fraction of remaining ET from base flow	none	2.11*E* − 02
AGWETP	Fraction of remaining ET from active GW	none	0.200
UZSN	Upper zone nominal soil storage	inches	1.807
INFTW	Interflow inflow parameter	none	10.000
IRC	Inflow recession constant	1/d	0.381

^1^Forest land; ^2^agricultural land; ^3^wetlands/water.

**Table 3:** General performance ratings for three evaluation criteria.

Performance rating	NSE		*R* ^2^		RE (%)	
	Monthly	Daily	Monthly	Daily	Monthly	Daily
Excellent	≥0.90	≥0.85	≥0.90	≥0.85	≤±5	≤±10
Very good	0.80–0.90	0.75–0.85	0.80–0.90	0.75–0.85	±5–±10	±10–±15
Good	0.70–0.80	0.65–0.75	0.70–0.80	0.65–0.75	±10–±15	±15–±25
Satisfactory	0.50–0.70	0.50–0.65	0.50–0.70	0.50–0.65	±15–±25	±25–±30
Unsatisfactory	<0.50	<0.50	<0.50	<0.50	≥±25	≥±30

**Table 4:** Model evaluated statistics for calibration period (2001–2004) and validation period (2005-2006).

Evaluation indicator	*R* ^2^	NSE	RE (%)
Calibration period (2001–2004)			
Monthly	0.97	0.97	−3.0%
Daily	0.86	0.86	−2.1%
Validation period (2005-2006)			
Monthly	0.96	0.94	−17.3%
Daily	0.81	0.80	−17.5%

**Table 5:** Relative error analysis for monthly mean flow data in calibration period in the Jiaoyi watershed (2001–2004).

Month	Observed (m^3^/s)	Simulated (m^3^/s)	RE (%)
1	4.15	4.31	3.90
2	3.56	2.78	−22.02
3	2.68	1.50	−44.03
4	2.90	0.43	−85.35
5	3.93	2.33	−40.78
6	6.24	6.43	2.96
7	57.28	61.78	7.84
8	75.77	75.55	−0.30
9	57.06	55.48	−2.77
10	20.06	16.33	−18.60
11	12.00	11.18	−6.90
12	8.82	8.53	−3.33

**Table 6:** Statistics of annual water balance components for agricultural land in Jiaoyi watershed (units: inches).

		Year							
		2001	2002	2003	2004	2005	2006	2007	Mean
Rainfall		26.99	22.38	39.75	37.13	42.19	26.06	38.0	33.21
Runoff	Surface flow	0.081	0.005	0.085	0.565	0.806	0.202	0.624	0.338
	Interflow	3.01	0.258	5.546	6.024	9.114	3.794	7.918	5.095
	Base flow	1.341	0.36	3.529	2.881	4.079	1.829	3.249	2.467
	Total	**4.432**	**0.623**	**9.16**	**9.47**	**14.0**	**5.825**	**11.79**	**7.9**

Deep groundwater		1.034	0.65	2.042	1.623	2.041	1.019	1.692	1.443
Evaporation	Potential	35.15	36.6	30.4	34.84	34.3	34.31	33.89	34.21
	Interception	4.193	3.371	5.37	5.02	4.626	2.927	3.844	4.193
	Upper zone	8.652	8.488	11.22	13.13	12.97	10.84	12.1	11.06
	Lower zone	6.53	8.208	5.261	6.432	5.486	6.951	3.783	6.093
	Ground water	1.035	1.514	0.968	1.272	0.894	1.12	0.418	1.032
	Base flow	0.821	0.344	1.205	1.364	1.335	0.969	1.407	1.064
	Total	**21.23**	**21.93**	**24.02**	**27.22**	**25.31**	**22.81**	**21.55**	**23.44**
